# 49193 The Association Between Specialized Pro-resolving Lipid Mediators and Markers of Neuroinflammation and Disease Severity in Acute Traumatic Brain Injury: A Prospective, Observational Cohort Study

**DOI:** 10.1017/cts.2021.646

**Published:** 2021-03-31

**Authors:** Roy Poblete

**Affiliations:** University of Southern California, Keck School of Medicine

## Abstract

ABSTRACT IMPACT: Characterizing specialized pro-resolving lipid mediators (SPMs) of inflammation in a cohort of adult patients with traumatic brain injury (TBI) will potentially identify novel biomarkers of disease and would generate hypotheses regarding the role of SPMs in the pathophysiology and recovery after brain injury OBJECTIVES/GOALS: Primary aim: evaluate the association between plasma and cerebrospinal fluid (CSF) SPM levels with inflammatory markers known to mediate blood brain barrier (BBB) disruption. Secondary aim: evaluate the association between SPM levels and disease severity, 14-day Glasgow Coma Scale score (GCS) and survival. METHODS/STUDY POPULATION: This is a single-center, prospective, observational cohort study (target N=50). Adult participants with moderate-to-severe non-penetrating TBI will have blood and CSF sampled serially for laboratory measures of SPMs (resolvins, protectins, lipoxins, maresins) and inflammatory peptides (TNF-α, IL-1β, IL-6, MMP-9, TIMP-1) by liquid chromatography’‘ mass spectrometry and RT-PCR & ELISA, respectively. Baseline patient characteristics, admission GCS, and 14-day GCS and mortality will be prospectively collected. To evaluate the association between SPMs and other continuous variables, Pearson’s and Spearman’s correlation will be used. Cox regression will be used to evaluate the association between SPM lipidomes and 14-day survival. RESULTS/ANTICIPATED RESULTS: As the primary outcome measure, the association between SPM levels with assayed inflammatory markers will be determined at 24 and 72 hours post-TBI. We hypothesize that SPMs will be negatively correlated with TNF-α, IL-1β, IL-6, MMP-9 and positively correlated with TIMP-1. As secondary outcome measures, the association between SPM levels with disease severity by admission GCS will be determined at 24 hours post-TBI, and the association between SPM levels and GCS and mortality will be determined at 14-days. We hypothesize that SPM levels will be positively associated with admission and 14-day GCS and will be independently associated with 14-day survival. DISCUSSION/SIGNIFICANCE OF FINDINGS: Using the SPM lipidome as a biomarker of disease is a novel tool for future translational research. It will inform a foundational mechanistic framework for TBI pathophysiology and attenuation of neuroinflammation post-TBI, providing rationale for pre-clinical and clinical research focused on novel therapeutics

